# Abortion care in post-decriminalization South Korea: the role of healthcare providers in advancing reproductive health rights

**DOI:** 10.4069/whn.2025.08.25.2

**Published:** 2025-09-30

**Authors:** Sunhye Kim

**Affiliations:** Department of Women’s Studies, Ewha Womans University, Seoul, Korea

## Introduction

In April 2019, the Constitutional Court of South Korea ruled that the provisions of the Criminal Act criminalizing abortion were unconstitutional and required the National Assembly to revise the law by the end of 2020 [1]. When the legislature failed to enact replacement legislation within the deadline, the penal provisions automatically lapsed on January 1, 2021. This historic legal shift meant that, for the first time since 1953, abortion was no longer a criminal offense. However, this change in legal status has not been accompanied by a corresponding transformation in healthcare provision. Abortion has not been integrated into the public health system as an essential reproductive health service, and the absence of statutory guidance, clinical protocols, and regulatory oversight has created a “healthcare vacuum.” Within this policy and institutional void, providers lack clear professional standards, and patients are left to navigate a fragmented, largely privatized service landscape. As a result, access to abortion in South Korea today is determined less by legal prohibition than by the availability, willingness, and interpretive discretion of individual healthcare providers.

Against this backdrop, this paper examines how abortion has functioned in South Korea, the social, legal, and policy contexts that led to the Constitutional Court’s decision to strike down the abortion ban, and the challenges that have emerged in the post-decriminalization landscape. It identifies key problems in current abortion service provision and discusses the roles healthcare providers should play in this evolving environment, alongside the structural and policy reforms required to integrate abortion into the public healthcare system. By situating these issues within broader legal, institutional, and sociocultural dynamics, the paper seeks to contribute to the advancement of reproductive health rights in South Korea and to inform strategies for ensuring equitable, stigma-free, and rights-centered abortion care.

## Abortion and reproductive health rights in South Korea: historical trajectories and current challenges

Abortion was first criminalized in South Korea under the 1953 Criminal Act. However, during the period of aggressive state-led family planning from the 1960s through the 1980s, aimed at reducing population growth, abortion was widely practiced and, in some cases, implicitly encouraged. The 1973 Mother and Child Health Act provided the legal basis for family planning programs and specified limited grounds for lawful abortion. Within this demographic policy framework, the abortion ban was often regarded as a “dead letter [2].” By the mid-2000s, as public discourse on a “low fertility crisis” intensified, the state shifted from population control to pronatalist policies and revived anti-abortion enforcement as part of this agenda [3]. This shift culminated in 2016, when the Ministry of Health and Welfare announced revisions to the Medical Service Act that increased penalties for physicians performing illegal abortions. The measure galvanized public attention and sparked widespread mobilization for the repeal of the Criminal Act’s abortion provisions. It marked a critical turning point, reframing abortion not merely as a moral controversy between “pro-choice” and “pro-life” camps but as an urgent reproductive health issue requiring systemic policy reform [4].

Internationally, reproductive health rights have been recognized as fundamental human rights under instruments such as the 1994 International Conference on Population and Development Programme of Action and the Convention on the Elimination of All Forms of Discrimination against Women. These define reproductive rights as encompassing the ability to decide freely and responsibly on the number, spacing, and timing of children, with access to the necessary information and means to do so, free from discrimination, coercion, and violence [5,6]. The World Health Organization likewise affirms that access to safe and legal abortion is an essential element of comprehensive reproductive health care, indispensable for protecting the health, rights, and lives of those who may become pregnant [7]. South Korea’s longstanding criminal restrictions raised significant concerns about its compliance with international treaty obligations and proved inadequate in safeguarding the health and rights of its population.

Despite the Constitutional Court’s landmark 2019 ruling striking down the abortion ban and the subsequent decriminalization that took effect in 2021, the integration of abortion into the public health system remains incomplete. Legal reform has established a symbolic guarantee of reproductive rights, but without parallel policy measures, institutional frameworks, and provider training, access to safe and timely abortion care continues to be shaped by structural gaps, medical gatekeeping, and uneven regional availability. This post-decriminalization moment, therefore, presents both opportunities and challenges in transforming reproductive health rights from a formal legal entitlement into a substantive reality.

## Post-decriminalization landscape: emerging issues and policy gaps

### Lack of a coordinated healthcare delivery system

The decriminalization of abortion in South Korea represented a historic legal shift, yet substantive progress toward realizing reproductive health rights has been limited. Although procedural codes exist within the healthcare system, no national clinical guidelines or standardized referral mechanisms specific to abortion care have been implemented, leaving service provision heavily dependent on the discretion of individual healthcare providers. In the absence of a coordinated framework, abortion services remain ad hoc, with wide variations in quality and scope. These gaps reflect the legacy of abortion’s longstanding treatment as a Criminal Act rather than as a legitimate component of healthcare, despite its clear medical nature. In the post-decriminalization context, dismantling abortion-related stigma is a necessary precondition for building a functional and equitable system, but progress in this area has been minimal.

### Barriers to access and exclusion from National Health Insurance

Financial barriers continue to be one of the most significant impediments to equitable abortion access. Because abortion is excluded from the National Health Insurance (NHI) benefits package, patients must pay the full cost of care—expenses that vary substantially by gestational age, provider, and facility type. Reports indicate that later-term abortions can require prohibitively high out-of-pocket payments, imposing a disproportionate burden on individuals with limited financial resources [8]. In some cases, these financial pressures have driven people to resort to unsafe or illegal methods, undermining both the intent of decriminalization and the constitutional guarantee of reproductive rights. Moreover, accurate and timely information about service availability is not systematically provided through public channels, forcing many to rely on informal networks or online sources that may be incomplete or outdated. Without NHI coverage and reliable public information, access to safe abortion remains inconsistent and insecure.

### Persistent legal ambiguities and outdated criteria

Furthermore, even though the grounds for lawful abortion under the Mother and Child Health Act lost their legal effect with the repeal of the Criminal Act’s abortion provisions, many clinics continue to invoke these outdated criteria as a basis for denying services. Such practices create unnecessary procedural hurdles and perpetuate confusion among both providers and patients. The persistence of obsolete legal norms highlights the enduring influence of prior criminalization on medical decision-making [9]. Looking ahead, a comprehensive legal framework that guarantees the full spectrum of sexual and reproductive health is urgently needed. However, policy debates remain disproportionately focused on punitive and regulatory approaches, raising concerns that opportunities to advance rights-based and patient-centered reproductive healthcare may be sidelined.

## The role of healthcare providers in advancing reproductive health rights

### Ensuring safe, legal, and equitable abortion care

Healthcare providers play a pivotal role in ensuring that abortion care is delivered safely, legally, and equitably. Experiences from various countries, including South Korea, demonstrate that providers—through their scientific authority and professional credibility—have effectively countered anti-rights narratives by supplying evidence-based medical information and concrete clinical experience, thereby shaping public debate and legislative processes [10]. Although legal uncertainty and the absence of clear guidelines can cause hesitancy or confusion, such conditions make the role of providers in advancing safe and lawful abortion care all the more critical. Even in contexts where abortion has been decriminalized, healthcare providers remain indispensable to ensuring that legal rights are translated into practical access, that professional standards are upheld, and that reproductive health rights are safeguarded.

### Building a professional infrastructure for rights-centered care

Key to advancing safe, respectful, and rights-centered abortion care is establishing a strong professional infrastructure led by healthcare providers. This requires embedding evidence-based competencies into medical and nursing curricula and reinforcing them through continuous professional development, ensuring that both current and future providers are able to deliver nonjudgmental care grounded in patient autonomy, informed consent, and confidentiality. These priorities align with the guidance of the International Federation of Gynecology and Obstetrics (FIGO), which affirms that access to safe abortion is an essential medical service integral to reproductive autonomy and human rights. FIGO further calls for comprehensive abortion care training—including medical and self-managed abortion, post-abortion care, and counseling—along with values-clarification workshops to reduce stigma and enhance provider readiness [11].

### Expanding roles and preparing for medical abortion

In addition to strengthening competencies, providers can lead institutional reforms by integrating abortion into NHI coverage, establishing referral networks, and developing national clinical guidelines. Such reforms should be grounded in collaboration among physicians, nurses, and allied health professionals to expand access and ensure consistent quality of care. With the anticipated approval of medical abortion, experiences from other countries show that the role of healthcare providers, particularly nurses, becomes increasingly significant in counseling, medication provision, and follow-up care alongside physicians [12]. Preparing for this shift requires clear supportive frameworks, targeted training, and coordinated practice models, making early action both urgent and necessary.

## Conclusion

This article has examined how, despite the repeal of Korea’s abortion ban, institutional voids, uneven service provision, and persistent stigma continue to restrict access to safe abortion care, and how the practices of healthcare providers shape both the possibilities and the limitations of this post-decriminalization landscape. Decriminalization was only the starting point; realizing reproductive autonomy requires embedding abortion within a supportive public health system and addressing the structural and cultural barriers that continue to limit access. Empowering providers as clinicians, advocates, and educators is therefore essential to ensuring equitable abortion care. Within this context, nurses play a particularly vital role, not only in direct clinical practice and patient counseling but also in advocacy efforts to reduce stigma, advance reproductive health rights, and educate patients and communities. By doing so, nurses help ensure that reproductive rights are realized as lived realities rather than remaining symbolic declarations.

## Appendix 1. Korean Summary of *“Abortion care in post-decriminalization South Korea: the role of healthcare providers in advancing reproductive health rights”*

한국의 ‘낙태죄’ 폐지 이후: 재생산 건강권 증진을 위한 의료인의 역할

김선혜 (교수, 이화여자대학교 여성학과)

2019년 헌법재판소가 형법상 ‘낙태죄’ 조항에 대해 헌법불합치 결정을 내렸으며, 기한 내 대안입법이 마련되지 않음에 따라 2021년 1월 해당 조항의 효력이 상실되었다. 이로써 임신중지는 더 이상 범죄로 처벌되지 않게 되었지만, 그럼에도 불구하고 공공보건 체계 안에서 필수적인 재생산 의료 서비스로 제도화되지 못한 채 남아 있다. 임상 지침과 국가적 전달 체계가 부재한 상황에서 접근성은 개별 의료인의 재량과 해석에 좌우되고 있으며 그 결과 임신중지 서비스는 여전히 사적이고 파편화된 시장에 의존한다. 여기에 경제적 부담, 지역 간 불균등, 법적 모호성, 사회적 낙인 등이 결합되면서 재생산 건강권은 실질적으로 보장되지 못하고 있다.

한국 사회에서 임신중지는 1953년 형법 제정 이후 범죄로 규정되었지만, 1960~80년대 가족계획 정책 아래에서는 국가가 사실상 이를 묵인하거나 장려한 역사가 있었다. 그러나 2000년대 이후 저출산 위기 담론이 강화되면서 ‘불법 인공 임신중절’의 단속과 규제가 다시 정책적 과제로 부상하였다. 이는 이제까지 임신중지가 여성의 건강과 권리보다는 국가의 인구정책 필요에 종속되어 왔음을 보여준다. 반면 국제인구개발회의(International Conference on Population and Development Programme of Action) 행동강령, 여성차별철폐협약(Convention on the Elimination of All Forms of Discrimination against Women), 세계보건기구(World Health Organization)의 권고가 분명히 밝히고 있듯이, 안전하고 합법적인 임신중지 접근은 재생산 건강권 보장의 핵심적 요소이다.

임신중지 비범죄화 이후 새롭게 부각되는 핵심 쟁점은 크게 네 가지다. 첫째, 국가 차원의 임상 지침과 연계 체계가 마련되지 않았다는 점, 둘째, 국민건강보험 적용이 제외되어 경제적 부담이 지속된다는 점, 셋째, 이미 효력을 상실했음에도 여전히 의료 현장에서 작동하는 모자보건법의 기준, 넷째, 임신중지를 사회적으로 주변화하는 낙인의 문제이다. 이러한 제약적 조건 속에서 임신중지의 실질적 접근성을 확대하기 위해 의료인이 담당해야 할 역할은 무엇보다 중요하다. 의료인은 단순히 시술 제공자에 머무르지 않고 환자의 권리를 존중하는 돌봄을 실천하며, 사회적 낙인을 완화하고 제도적 변화를 주도하는 옹호자이자 교육자로서 핵심적인 역할을 수행한다. 이를 위해 의학 교육과 간호 교육 과정에는 근거에 기반한 임상 역량뿐 아니라 임신중지에 대한 자신의 가치관과 태도를 성찰하고 조정하는 훈련이 포함되어야 하며, 이를 바탕으로 지속적 전문 교육이 강화될 필요가 있다. 또한 향후 약물적 임신중지 도입에 대비해 의사와 간호사가 협력하는 다층적 서비스 체계를 마련하고 특히 간호사가 상담·약물 제공·사후 관리에 적극적으로 참여할 수 있도록 제도적 기반을 구축해야 한다.

결론적으로 재생산 건강권 보장을 위한 ‘낙태죄’ 폐지는 출발점에 불과하다. 임신중지가 필수 의료 서비스로서 공공보건 체계 안에 자리매김하기 위해서는 제도적 통합과 경제적·사회적 장벽의 해소가 필수적이다. 더불어 의료인을 임상가이자 옹호자, 교육자로 역량을 강화하는 것은 권리 기반의 임신중지 돌봄을 실현하는 핵심 경로이며, 특히 간호인은 상담과 교육, 사회적 옹호 활동을 통해 재생산권을 단순한 선언적 권리가 아닌 일상 속에서 실현되는 권리로 만드는 데 중요한 역할을 담당한다.

* This Korean Summary is based on the following Issues and Perspectives manuscript, for Korean readers.

*“Abortion care in post-decriminalization South Korea: the role of healthcare providers in advancing reproductive health rights”* (Womens Health Nurs 2025;31(3):165-169. https://doi.org/10.4069/whn.2025.08.25.2).
